# Headache, depression and anxiety: associations in the Eurolight project

**DOI:** 10.1186/s10194-016-0649-2

**Published:** 2016-06-01

**Authors:** Christian Lampl, Hallie Thomas, Cristina Tassorelli, Zaza Katsarava, Jose Miguel Laínez, Michel Lantéri-Minet, Daiva Rastenyte, Elena Ruiz de la Torre, Lars Jacob Stovner, Colette Andrée, Timothy J. Steiner

**Affiliations:** Headache Medical Center, Linz, Austria; Department of Neurogeriatric Medicine and Remobilisation, Hospital of the Sisters of Charity, Linz, Austria; Department of Neuroscience, Norwegian University of Science and Technology, Edvard Griegs Gate, Trondheim, Norway; Headache Science Centre, C Mondino National Neurological Institute, Pavia, Italy; Department of Brain and Behavioural Sciences, University of Pavia, Pavia, Italy; Department of Neurology, University of Duisberg-Essen, Essen, Germany; Department of Neurology, Evangelical Hospital Unna, Unna, Germany; Department of Neurology, Hospital Clinico Universitario, University of Valencia, Valencia, Spain; Departement d’Evaluation et Traitement de la Douleur, Centre Hospitalo-Universitaire de Nice, Nice, France; INSERM/UdA, U1107, Neuro-Dol, Clermont-Ferrand, France; Lithuanian University of Health Sciences, Kaunas, Lithuania; Asociacion Española de Pacientes con Cefalea (AEPAC), Valencia, Spain; Norwegian Advisory Unit on Headache, St Olavs University Hospital, Trondheim, Norway; Department of Pharmaceutical Sciences, University of Basel, Basel, Switzerland; Department of Population Health, Luxembourg Institute of Health, Strassen, Luxembourg; Division of Brain Sciences, Imperial College London, London, UK

**Keywords:** Headache, Migraine, Tension-type headache, Medication-overuse headache, Depression, Anxiety, Comorbidity, Associations, Public health, Europe, Eurolight project, Global Campaign against Headache

## Abstract

**Background:**

Headache disorders and psychiatric disorders are both common, while evidence, mostly pertaining to migraine, suggests they are comorbid more often than might be expected by chance. There are good reasons for establishing whether they are: symptoms of comorbid illnesses may summate synergistically; comorbidities hinder management, negatively influencing outcomes; high-level comorbidity indicates that, where one disease occurs, the other should be looked for. The Eurolight project gathered population-based data on these disorders from 6624 participants.

**Methods:**

Eurolight was a cross-sectional survey sampling from the adult populations (18–65 years) of 10 EU countries. We used data from six. The questionnaire included headache-diagnostic questions based on ICHD-II, the Headache-Attributed Lost Time (HALT) questionnaire, and HADS for depression and anxiety. We estimated odds ratios (ORs) to show associations between migraine, tension-type headache (TTH) or probable medication-overuse headache (pMOH) and depression or anxiety.

**Results:**

pMOH was most strongly associated with both psychiatric disorders: for depression, ORs (*vs* no headache) were 5.5 [2.2–13.5] (*p* < 0.0001) in males, 5.5 [2.9–10.5] (*p* < 0.0001) in females; for anxiety, ORs were 10.4 [4.9–21.8] (*p* < 0.0001) and 7.1 [4.5–11.2] (*p* < 0.0001). Migraine was also associated with both: for depression, ORs were 2.1 [1.3–3.4] (*p* = 0.002) and 1.8 [1.1–3.1] (*p* = 0.030); for anxiety 4.2 [2.8–6.3] (*p* < 0.0001) and 2.4 [1.7–3.4] (*p* < 0.0001). TTH showed associations only with anxiety: ORs 2.5 [1.7–3.7] (*p* < 0.0001) for males, 1.5 [1.1–2.1] (*p* = 0.021) for females. Participants with migraine carried 19.1 % probability of comorbid anxiety, 6.9 % of depression and 5.1 % of both, higher than the representative general-population sample (14.3, 5.6 and 3.8 %). Probabilities in those with MOH were 38.8, 16.9 and 14.4 %; in TTH, they did not exceed those of the whole sample. Comorbid psychiatric disorder did not add to headache-attributed productive time losses, but weak associations existed (*R*^*2*^ = 0.020–0.082) for all headache types between lost productive time and probabilities of depression and, less so, anxiety.

**Conclusion:**

In this large study we confirmed that depression and especially anxiety are comorbid more than by chance with migraine, and showed the same is true, but more strongly, with MOH. Arguably, migraine patients and, more certainly, MOH patients should be screened with HADS in pursuit of best outcomes.

## Background

Headache disorders are very common. In the Global Burden of Disease Study 2010 (GBD2010), tension-type headache (TTH) and migraine were found to be the second and third most prevalent disorders in the world [1]. Additionally, the group of disorders characterised by headache recurring on ≥15 days/month affects 1.7–4 % of the world’s adult population [2], many of them having medication-overuse headache (MOH) [3]. Headache disorders are also disabling: in GBD2013, they were revealed as the third cause of disability worldwide [4, 5], migraine and MOH being the substantial contributors. Also in GBD2013, depression and anxiety, both common psychiatric disorders, were ranked second and ninth highest causes of disability worldwide [4].

Common disorders occur together (are comorbid) by chance. Depression and anxiety are comorbid with each other more than by chance [6]. In addition, several studies point to a higher probability (2 to 4 times) of psychiatric disorders among people with migraine [7–12]. Few studies have specifically considered TTH, although people with episodic TTH were found no more likely than controls to experience anxiety or mood disorders [13], while those among a Chinese elderly population with chronic TTH were twice as likely to be suffering from depression [14]. There is evidence associating psychiatric morbidity with medication overuse [15], but no data on psychiatric comorbidity with MOH in the general population.

There are good reasons for wishing to establish whether or not there are associations between the common headache disorders and the common psychiatric disorders, all of them major contributors to public ill health and the burden of disability. The symptoms of comorbid illnesses are expected to summate synergistically. Comorbidities hinder management and negatively influence outcomes, more so when they are unrecognized. Comorbidity occurring more than by chance indicates that, where one disease occurs, the other should be looked for. It also suggests causal relationships or common aetiological factors, which should be elucidated.

The Eurolight project was an initiative supported by the European Commission Executive Agency for Health and Consumers (EHAC), and a partnership activity within the Global Campaign against Headache conducted by *Lifting The Burden* (LTB), a UK-registered non-governmental organization in official relations with the World Health Organization. Eurolight gathered data on these disorders from over 9000 variously-selected adult participants in a questionnaire-based cross-sectional survey conducted in 10 countries in Europe [16]. The survey included demographic enquiry and screened for headache, depression and anxiety, with diagnostic questions for migraine, TTH and MOH. We analysed the data for evidence of associations between depression and anxiety and headache type, by gender.

## Methods

The methods of the Eurolight project have been described in detail elsewhere [17], and are summarised here.

### Ethics

The National Ethics Committee of Luxembourg gave overall approval of the protocol and provisions for data protection. Further approvals were obtained from national and/or local ethics committees wherever needed as the methods for recruitment of participants differed between countries. In every country, prospective participants received written information explaining the project and its purpose.

### Project design and sampling

Eurolight was a cross-sectional survey conducted from November 2008 to August 2009. It used modified cluster sampling in 10 countries which together represented >60 % of the adult population (18–65 years) of the European Union: Austria, France, Germany, Ireland, Italy, Lithuania, Luxembourg, Netherlands, Spain and United Kingdom (UK). The sampling methods varied between countries according to what was feasible, and are fully described elsewhere [17]. They are summarised in Table 1 for the six countries contributing to this analysis, which are those with samples drawn from the general population. In three other countries (Austria, France and UK), sampling was to some extent patient-based (although not headache patients), while additional samples from Spain and the Netherlands, and the only sample from Ireland, were recruited through patients’ organisations [17]. None of these was included in this analysis because of the biases inherent in them.

**Table 1:** Summary of data collection methods, and demographics of samples, in each country

Country	Sampling and data collection method	Questionnaires distributed and returned	Gender	n	Age (years)
		N/n			(mean ± SD)
Germany	Random urban (50 %) and rural (50 %) samples aged 18–65 years from general population listings supplied by local municipal authority. Questionnaires distributed and returned by post. No reminders sent.	3000/318	M	136	45.7 ± 13.4
			F	182	43.8 ± 11.8
Italy	Random urban (70 %) and rural (30 %) samples drawn from general population using listings supplied by Azienda Sanitaria Locale of Pavia, stratified with regard to gender, age (in range 18–65 years) and education. Questionnaires distributed and returned by post. No reminders sent.	3500/487	M	203	44.9 ± 12.9
			F	284	42.4 ± 12.3
Lithuania	Sample drawn from Kaunas city and Kaunas region using Residents’ Register Service, reflecting age (in range 18–65 years) and gender composition of Lithuania and proportions living in rural (33 %) or urban (67 %) areas. Data collection face-to-face, conducted by medical students “cold-calling” door-to-door.	1137/573	M	237	39.8 ± 14.0
			F	336	41.7 ± 13.7
Luxemburg	Sample aged 18–65 years, stratified for age, gender, region and nationality, drawn from general population via national social security registry (IGSS). Questionnaires distributed and returned by post. Reminders sent one month later to non-responders.	6498/1833	M	769	40.8 ± 12.8
			F	1064	40.3 ± 12.6
Netherlands	Survey conducted by market research company with access to population sample of 200,000, representative with regard to gender, age (in range 18–65 years), region and education. Questionnaire distributed by internet, to be completed on-line. Study stopped when >2000 received back.	unknown/2414	M	1214	43.7 ± 13.5
			F	1200	41.6 ± 12.9
Spain	Random sample of employees of companies operating in national postal services in 10 areas of Spain, stratified to be representative of general working population with regard to gender, age (in range 18–65 years) and education. Ten occupational health physicians delivered and took return of questionnaires. One telephone reminder to non-responders.	1700/999	M	410	44.1 ± 11.9
			F	589	41.7 ± 11.8
All		unknown/6624	M	2969	42.9 ± 13.2
			F	3655	41.4 ± 12.6
			All	6624	42.1 ± 12.9

### Instruments

The survey used the same structured questionnaire in all countries [18], a derivative of LTB’s HARDSHIP questionnaire developed for population-based surveys [19]. It was translated into the local languages following LTB’s translation protocol for lay documents [20]. It had multiple parts. Demographic questions were followed by screening questions for headache and, in those screening positively, by headache-diagnostic questions based on ICHD-II [21]. The timeframe for enquiry was the preceding year. Participants identifying more than one headache type were asked to report only on the one that was most bothersome. Diagnoses were made by computerized algorithm [19]. The algorithm first identified, and separated, participants reporting headache on ≥15 days/month, of whom additional questions enquired into medication use. Probable MOH (pMOH) was diagnosed when (a) duration was typically >4 h and (b) frequency of acute medication use was ≥15 days/month and the medication was simple analgesics only, or ≥10 days/month when it was any other (compound analgesics, opioids, triptans and/or ergots). A diagnosis of pMOH trumped all other diagnoses. The remainder of this group were diagnosed as “other headache on ≥15 days/month”. To all others (with headache on <15 days/month), the algorithm applied ICHD-II criteria for migraine, TTH, probable migraine and probable TTH in that order. In the analyses, migraine and probable migraine were considered together, as were TTH and probable TTH [22, 23].

To enquire into impact, the questionnaire imported LTB’s Headache-Attributed Lost Time (HALT) Index as a module. HALT captured lost productive time (days lost completely, and days of <50 % productivity) from paid and household work because of headache, and missed social and leisure activities [24]. In the analysis of HALT data, days of <50 % productivity were counted as and added to wholly lost days, being counterbalanced by days that were affected by headache but with productivity still >50 %, which were ignored. Lost paid workdays and lost household workdays were summed separately, and then totalled for lost productive time.

To assess anxiety and depression among participants, the questionnaire imported, as a further module, the Hospital Anxiety and Depression Scale (HADS) [25]. This screening instrument was preferred to more specific instruments: firstly, it is easily applied in surveys of this type; secondly – and more importantly – it detects the subjective manifestations of anxiety and depression rather than somatic symptoms of distress [25], which, confoundingly, might include headache. HADS consists of two subscales, HADS-Anxiety (HADS-A) and HADS-Depression (HADS-D), each of seven items. In response to each item, participants report their subjective experience during the preceding week, rating it 0–3 (3 indicating maximum symptom severity). The sum of each subscale has a potential range of 0–21. As recommended in the original description [25], we took a threshold of 11 on the respective subscale to indicate caseness for anxiety or depression.

### Statistics

Categorical variables are described in terms of frequency (n) and proportions (%). Continuous variables are described in terms of means ± standard deviations (SDs).

We calculated, as percentages with 95 % confidence intervals (CIs), 1-year prevalences of migraine and TTH and point prevalences of pMOH and other headache on ≥15 days/month in the participating sample, overall and by gender. We calculated point prevalences of depression and anxiety, and of comorbid depression plus anxiety, in the participating sample overall, by gender and by headache type. We performed logistic regressions, stratified by gender, in order to explore associations between all headache and headache types on the one hand and depression and anxiety on the other, taking the latter two as the dependent variables. We calculated odds ratios (ORs) with 95 % CIs.

To establish whether depression or anxiety influenced the impact of headache on lost productive time, we did the following. For each headache type, we plotted headache-attributed lost time (measured by HALT and treated as continuous data) against HADS-D and HADS-A scores (also continuous data), and performed simple regression analyses, calculating R^2^ for the linear trendlines. We also conducted this analysis in reverse, with depression or anxiety rather than days lost as dependent variables.

We performed analyses using Microsoft Excel:mac 2011 software version 14.5.7.

## Results

### Demographics

Eurolight collected 9269 correctly completed questionnaires from all 10 countries, but the population-based sample from six countries included 6624 participants (males 2969 [44.8 %]; females 3655 [55.2 %]; mean age 42.1 ± 12.9 years) (Table 1).

### Prevalences

The prevalences of each headache type in this sample, overall and by gender, are shown in Table 2. TTH (39.4 %) was the most prevalent headache type, but only a little more so than migraine (35.9 %). Both migraine (1.6:1) and pMOH (2.8:1), but not TTH (0.9:1) were more prevalent among females.

**Table 2:** Prevalences of each headache type in the sample, overall and by gender

Gender	Prevalences (%) [95 % CI]			
	Migraine	Tension-type headache	Probable medication-overuse headache	Other headache on ≥15 days/month
All (*N* = 6624)	35.9 [34.7–37.1]	39.4 [38.2–40.6]	3.0 [2.6–3.4]	2.5 [2.1–2.9]
Male (*n* = 2969)	26.9 [25.3–28.5]	41.6 [39.8–43.4]	1.5 [1.1–1.9]	2.6 [2.0–3.2]
Female (*n* = 3655)	43.1 [41.5–44.7]	37.7 [36.1–39.3]	4.2 [3.5–4.9]	2.3 [1.8–2.8]

The prevalences of depression and anxiety, overall, by gender and by headache type (the last indicating comorbidities between these disorders), are shown in Table 3. Anxiety in the total sample was 2.5 times more prevalent than depression. This ratio was reduced to 1.7:1 in the subsample with no headache. Both disorders were more prevalent among females: depression by a ratio of 1.2:1 (*p* = 0.0406), anxiety more so, by 1.8:1 (*p* < 0.0001; Fisher’s exact test).

**Table 3:** Prevalences of depression and anxiety in the sample, overall, by gender and by headache type

	Prevalences (%) [95 % CI]		
	Depression	Anxiety	Depression + anxiety
All (*N* = 6624)	5.6 [5.0–6.2]	14.3 [13.7–15.1]	3.8 [3.3–4.3]
Gender			
Male (*n* = 2969)	4.9 [4.1–5.7]	9.9 [8.8–11.0]	2.9 [2.3–3.5]
Female (*n* = 3655)	6.1 [5.3–6.9]	17.8 [16.6–19.0]	4.6 [3.9–5.3]
Headache type			
No headache (*n* = 1271)	3.5 [2.5–4.5]	6.1 [4.8–7.4]	1.7 [1.0–2.4]
Migraine (*n* = 2375)	6.9 [5.9–7.9]	19.1 [17.5–20.7]	5.1 [4.2–6.0]
TTH (*n* = 2613)	4.5 [3.7–5.3]	12.1 [10.9–13.4]	3.0 [2.3–3.7]
pMOH (*n* = 201)	16.9 [11.7–22.1]	38.8 [32.1–45.5]	14.4 [9.5–19.3]
Other headache on ≥15 d/m (*n* = 164)	3.6 [0.8–6.4]	12.1 [7.1–17.1]	1.8 [0–3.8]

*TTH* tension-type headache, *pMOH* probable medication-overuse headache, *d/m* days/month

### Comorbidity

Depression and anxiety were highly comorbid with each other in the total sample (3.8 % against an expectation of 0.8 % from chance association alone).

In comparison with the total sample, depression and anxiety were each comorbid more than by chance with both migraine and pMOH but not with TTH or other headache on ≥15 days/month. However, the lower part of Table 3 indicates that anxiety was more prevalent in those with any of the headache types than in those with no headache. Depression was more prevalent in all but other headache on ≥15 days/month, although in TTH the excess was not significant. We confirmed these associations by calculating ORs, which are presented in Table 4. As predicted by the data in Table 3, the associations with pMOH were very strong. The analyses suggested that all associations, except that between depression and pMOH, were stronger in males than in females.

**Table 4:** Associations between headache types and depression and anxiety, by gender

Headache type	Odds ratios [95 % CI] *vs* no headache as reference							
	Depression				Anxiety			
	Males	p	Females	p	Males	p	Females	p
Migraine	2.1 [1.3–3.4]	0.002	1.8 [1.1–3.1]	0.030	4.2 [2.8–6.3]	<0.0001	2.4 [1.7–3.4]	<0.0001
Tension-type headache	1.1 [0.7–1.8]	0.597	1.3 [0.8–2.3]	0.328	2.5 [1.7–3.7]	<0.0001	1.5 [1.1–2.1]	0.021
Probable medication-overuse headache	5.5 [2.2–13.5]	<0.0001	5.5 [2.9–10.5]	<0.0001	10.4 [4.9–21.8]	<0.0001	7.1 [4.5–11.2]	<0.0001
Other headache on ≥15 days/month	1.9 [0.7–5.7]	0.242	0.6 [0.1–2.9]	0.568	4.7 [2.1–9.7]	0.0001	1.3 [0.6–2.6]	0.474

**Fig. 1:**
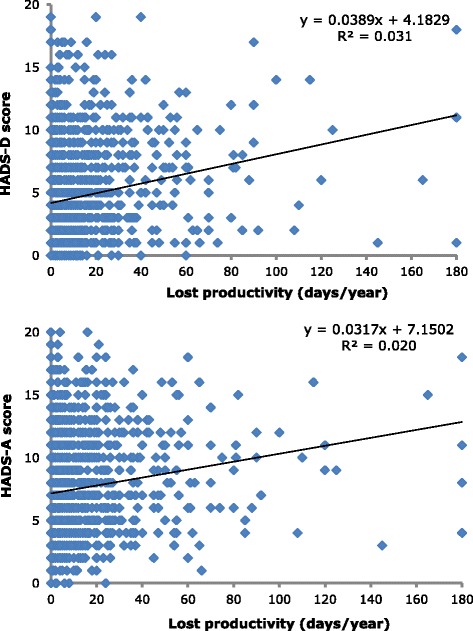
Illustrative plots with linear trendlines of HADS-D score (*top*) and HADS-A score (*bottom*) against headache-attributed lost time in participants with migraine

To enquire into how comorbid depression or anxiety influenced the impact of headache on lost productive time, we plotted headache-attributed lost time (measured by HALT) against HADS-D and HADS-A scores. We did this for migraine, TTH and pMOH, but found no influence when treating days lost as the dependent variable (R^2^ in the range 0.0005–0.01). However, with the analyses reversed (depression and anxiety as dependent variables), weak influences were detected in all cases: most strongly between pMOH and depression (*R*^*2*^ = 0.082) and pMOH and anxiety (*R*^*2*^ = 0.059); next most strongly between TTH and depression (*R*^*2*^ = 0.036) and migraine and depression (*R*^*2*^ = 0.031), and least strongly between TTH and anxiety (*R*^*2*^ = 0.026) and migraine and anxiety (*R*^*2*^ = 0.020). The relationships with migraine are shown in Fig. 1 to illustrate.

## Discussion

The key findings were (1) a confirmation that anxiety in particular, but also depression, are comorbid more than is expected by chance with migraine, and (2) first clear evidence of similar but stronger relationships with pMOH. Anxiety but not depression was weakly comorbid with TTH.

Before we comment further on these findings, we need to recall the limitations of the Eurolight study: the full study was not entirely population-based, sampling methods differed between countries [17], and participation rates were very low in some [16]. We were selective here, analysing data only from samples drawn from the general population, but even so we cannot argue that the estimates of *prevalence* that we made use of were reliable. Eurolight was a study of headache impact rather than prevalence [16], and this is no doubt reflected in the reported migraine prevalence of 35.9 %, which is much higher than the global and European means [1, 26] and only a little below that of TTH (39.4 %). The reported prevalence of pMOH (3.0 %) was also on the high side [3]. It was not, of course, a purpose of this study to report prevalences of headache types, but we draw attention at the outset to this potential source of bias.

As for the prevalence estimates for psychiatric disorders, we found that depression affected about one adult in 20 (5.6 %), while anxiety was almost three times as common (14.3 %). Both disorders were more prevalent in females, but this gender association was much stronger with anxiety. Recent reviews have estimated the global prevalence of depression in the range 4.4–5.0 % [27] and of anxiety in the range 4.8–10.9 % [28], with national studies generally finding these disorders to be more prevalent in developed Western countries [29, 30]. Therefore our estimates were in keeping with expectations, while towards their upper limits. It should be noted that HADS is a screening rather than diagnostic instrument for these disorders [25], with a tendency to underestimate prevalence of both [6]; we conclude, therefore, that levels of psychopathology in the sample, especially anxiety, were high. Depression and anxiety were highly comorbid with each other, as is invariably reported [6, 31].

We found, also, comorbidity at levels greater than expected by chance between each psychiatric disorder and each of migraine and pMOH. This, almost certainly, is the explanation of the high levels of psychopathology in our sample, especially since, on the psychiatric side, the associations were stronger with anxiety. On the headache side, the associations were strongest with pMOH, but still strong with migraine. Only anxiety showed an association with TTH, and this was weak. Eurolight diagnosed only the most bothersome headache type in participants reporting more than one [18, 22]; most people with both migraine and TTH would describe the former as more bothersome and not report the latter. While the prevalence of TTH might as a result be underestimated [22], this could not have masked an association between depression and TTH uncomplicated by migraine.

Multiple studies have earlier found increased prevalences of depression and anxiety in people with migraine relative to those without headache [12, 32–34]. Migraine and anxiety are clearly comorbid, and several studies have demonstrated, as we did, that this association is stronger than that between migraine and depression [11, 35, 36]. However, relatively few population-based data exist on psychiatric comorbidity with TTH. Merikangas found no increased comorbidity between either depression or anxiety and TTH in a Swiss study [7]; we were almost in agreement with her. On the other hand, the very large Norwegian HUNT study found that both migraine and non-migrainous headache (80 % of the latter being TTH) were comorbid with anxiety and depression (36).

For all headache types, these relationships became more pronounced with increasing headache frequency. The spectrum of highly-frequent headache (occurring on ≥15 days/month) includes chronic migraine, chronic TTH and MOH. Eurolight did not attempt to diagnose the first two, which cannot be done reliably in such surveys [22], but we found strong associations with pMOH: ORs of 5.5 for depression and 7–10 (greater in males) for anxiety. Until now, no population-based studies have specifically assessed psychiatric comorbidity with MOH, although in clinical samples MOH patients frequently exhibit depression and anxiety [37]. It has also been shown that depression and anxiety are risk factors for developing MOH in migraineurs [38], so comorbidity is not surprising. Nevertheless, this study is the first to show it.

Most of these associations showed some evidence of a gender-relationship, being stronger in males. Although individual differences were not significant, the consistency is striking. Victor et al., in a US study, observed that males with migraine were more likely than females with migraine to report anxiety or depressive symptoms compared with the same gender without headache [33]. However, a much earlier US study had found no gender difference in comorbidity between migraine and major depression or anxiety disorders [39]. If such a difference should exist, we would presume that the socio-environmental and/or genetic factors explaining these comorbidities do not play exactly the same roles in males as in females.

On a clinical level, the importance of these associations is in how they might influence management. The key question is: should a physician treating a patient complaining of headache screen for comorbid psychiatric disorder? The data in the lower part of Table 3 indicate the probabilities of comorbid depression or anxiety by headache type, and are likely to be applicable to patients typically seen in primary care rather than those who have found their way to specialised clinics. A patient with migraine has a 19 % probability of comorbid anxiety, almost 7 % of depression and 5 % of both. These are certainly higher, but not dramatically so, than the 14.3, 5.6 and 3.8 % in the entire sample representative of the general population. On the other hand, comorbid psychiatric disorder, when present, adds to overall morbidity and, if not identified and itself treated, leads to unsuccessful headache management, or at least a poorer outcome. This suggests that, even in primary care where time is at a premium, there is a case for screening migraine patients with HADS. Against this it may be argued that, if anxiety – the more common comorbid disorder by a large margin – is actually secondary to the worrying headache symptoms that have brought the patient into a consultation, then treating the headache is the priority and perhaps all that is necessary. We have recently shown that interictal anxiety is an important component of the burden of migraine [40]. In a patient with MOH, the probability of comorbid anxiety is almost 39 %, of depression 17 % and of both 14 %, which make the case for screening with HADS considerably more compelling. In TTH, the probabilities do not exceed those of the whole sample. In all cases, probabilities are higher in females than males, but this reflects the underlying prevalences of these disorders.

On a public-health level, our interest was more in how the effects of these comorbid disorders might summate. We assumed that a psychiatric disorder, when present, would add to overall morbidity and that this would be evident in the HALT analysis. We found, instead, that comorbid anxiety or depression did not add significantly to lost productive time attributable to headache. But there was a weak association – with all headache types, but most strongly pMOH – between lost productive time and the probability of depression or, less so, anxiety. We can speculate that this indicated cause: that comorbid anxiety and depression were at least in part the consequences of increasing lost productive time (or of the symptoms causing it), rather than due to underlying biological susceptibility. We have no further evidence in support of this idea.

While the Eurolight study had a number of methodological limitations, mentioned earlier, it also had several strengths. It was a European community-wide survey, sampling from ten countries [17], of which we took six (from west, north, east, central and south Europe) that provided population-based data. It used a validated questionnaire (HARDSHIP) diagnosing headache types by applying modified ICHD-II criteria algorithmically [19] and a widely accepted screening instrument (HADS) for psychiatric disorders [25]. It is, for the time being, the only source of population-based gender-specific data on psychiatric comorbidity with TTH and MOH.

## Conclusion

In this large cross-sectional European study, we confirmed that depression and especially anxiety are comorbid more than is expected by chance with migraine. We found the same, but more strongly, with pMOH, and that anxiety but not depression is weakly comorbid with TTH. There is an arguable case for screening migraine patients with HADS, and a stronger case for MOH patients, in order to achieve best outcomes. Comorbid anxiety or depression did not add to lost productive time, but weak associations existed in all headache types between lost productive time and the probability of depression or, less so, anxiety.
